# New Continuous Process for the Production of Lipopeptide Biosurfactants in Foam Overflowing Bioreactor

**DOI:** 10.3389/fbioe.2021.678469

**Published:** 2021-05-28

**Authors:** Jean-Sébastien Guez, Antoine Vassaux, Christian Larroche, Philippe Jacques, François Coutte

**Affiliations:** ^1^Université Clermont Auvergne, Clermont Auvergne INP, CNRS, Institut Pascal, Clermont-Ferrand, France; ^2^Université de Lille, UMRt BioEcoAgro 1158-INRAE, équipe Métabolites Secondaires d’origine Microbienne, Institut Charles Viollette, Lille, France; ^3^Université de Liège, UMRt BioEcoAgro 1158-INRAE, équipe Métabolites Secondaires d’origine Microbienne, TERRA Teaching and Research Centre, MiPI, Gembloux Agro-Bio Tech, Gembloux, Belgium; ^4^Lipofabrik, Polytech-Lille, Cité Scientifique, Villeneuve d’Ascq, France

**Keywords:** continuous culture, foaming process, overflowing culture, lipopeptide antibiotic, mycosubtilin, biosurfactant, *Bacillus subtilis*, continuous product removal

## Abstract

In this work, an original culture process in bioreactor named overflowing continuous culture (O-CC) was developed to produce and recover continuously mycosubtilin, a lipopeptide antifungal biosurfactant of major interest. The lipopeptide production was first investigated in shake conical flasks in different culture media [ammonium citrate sucrose (ACS), Difco sporulation medium (DSM), and Landy], followed by a pH condition optimization using 3-(*N*-morpholino)propanesulfonic acid (MOPS) and 2-(*N*-morpholino)ethanesulfonic acid (MES) buffered media. A simple theoretical modeling of the biomass evolution combined with an experimental setup was then proposed for O-CC processed in stirred tank reactor at laboratory scale. Seven O-CC experiments were done in modified Landy medium at the optimized pH 6.5 by applying dilution rates comprised between 0.05 and 0.1 h^–1^. The O-CC allowed the continuous recovery of the mycosubtilin contained in the foam overflowing out of the reactor, achieving a remarkable *in situ* product removal superior to 99%. The biomass concentration in the overflowing foam was found to be twofold lower than the biomass concentration in the reactor, relating advantageously this process to a continuous one with biomass feedback. To evaluate its performances regarding the type of lipopeptide produced, the O-CC process was tested with strain BBG116, a mycosubtilin constitutive overproducing strain that also produces surfactin, and strain BBG125, its derivative strain obtained by deleting surfactin synthetase operon. At a dilution rate of 0.1 h^–1^, specific productivity of 1.18 mg of mycosubtilin⋅g^–1^(DW)⋅h^–1^ was reached. Compared with other previously described bioprocesses using almost similar culture conditions and strains, the O-CC one allowed an increase of the mycosubtilin production rate by 2.06-fold.

## Introduction

Thousands of microbial bioactive products originate from modular multienzymatic proteins either from polyketide synthase (PKS), as erythromycins and tetracyclines; from non-ribosomal peptide synthetase (NRPS), as penicillins, vancomycins, surfactins, fengycins, and cephalosporins; or from hybrid PKS/NRPS, as daptomycins, epothilones, or mycosubtilins (Fischbach and Walsh, 2006; Sattely et al., 2008). Depending on their wide structural diversity, these small compounds exhibit a wide range of biological activities and chemical properties: antibacterial, antifungal, iron chelating, hemolytic, immunosuppressive, antitumoral, and surfactants. Mycosubtilin belongs to the iturin family, which also includes iturins A, A_*L*_, C, D, and E; bacillomycins D, F, L, and Lc; and bacillopeptin (Jacques, 2011). Structurally, mycosubtilin has a β-amino fatty acid linked to a heptapeptide moiety. Mycosubtilin exhibits several isoforms according to the length (C-15 to C-18) and the isomery (linear, *iso*, and *anteiso*) of the fatty acid (Ongena and Jacques, 2008; Béchet et al., 2013). Like the other lipopeptides, mycosubtilin contains a hydrophobic tail and a hydrophilic head increasing foaming and dropping surface tension (Razafindralambo et al., 1998). Mycosubtilin shows a wide spectrum of antifungal activities against several pathogen yeasts such as *Candida albicans* and *Cryptococcus neoformans* (Fickers et al., 2009); foodborne pathogens such as *Candida krusei*, *Paecilomyces variotii*, and *Byssocchlamys fulva* (Kourmentza et al., 2021); and different plant fungal pathogens such as *Botrytis cinerea*, *Fusarium oxysporum*, *Pythium aphanidernatum*, *Venturia inaequalis*, *Bremia lactucae*, and *Zymoseptoria tritici* (Leclere et al., 2005; Deravel et al., 2014; Farace et al., 2015; Mejri et al., 2018; Mihalache et al., 2018; Desmyttere et al., 2019); and it displays low ecotoxicity profile compared with conventional pesticides (Deravel et al., 2014). The direct antagonism of mycosubtilins against these pathogens, with low values of MIC ranging from 2 to 32 μM, is strongly related to the type of mycosubtilin isoform (Fickers et al., 2009; Béchet et al., 2013). The authors also highlighted the ability of mycosubtilin to trigger an immune response in plants (Farace et al., 2015). This property to induce systemic resistance of plants opens a new field of industrial applications. However, the use of mycosubtilin mixtures potentially requires large amounts of product (Mnif and Ghribi, 2015), particularly in the field of pharmaceutics/cosmetics (kilograms) or plant disease control (tons).

Consequently, the development of economically viable industrial bioprocesses becomes necessary. By now, the high production costs of lipopeptides and the low production yields have been identified as major bottlenecks (Inès and Dhouha, 2015; Beltran-Gracia et al., 2017). To face these obstacles, a first efficient approach is to lower the cost of the substrate by targeting cheap raw materials and optimizing culture media composition (Das and Mukherjee, 2007; Mizumoto and Shoda, 2007; Wang et al., 2008; Marti et al., 2015; Dang et al., 2019; Xu et al., 2020). A complementary approach is to increase lipopeptide productivity of the natural biocatalysts with the screening of natural strains or with applying metabolic and genetic engineering strategies (Leclere et al., 2005; Béchet et al., 2013; Coutte et al., 2015; Dang et al., 2019; Wu et al., 2019; Xu et al., 2020). As the biological activity of the lipopeptide mixture strongly depends on the lipopeptide family or isoform produced, the selectivity of the production was also addressed within several studies of environmental parameters as oxygen transfer (Guez et al., 2008; Fahim et al., 2012), temperature (Fickers et al., 2008), and amino acid feeding (Fickers et al., 2009). The pH is also one of the main parameters to consider, as it was previously demonstrated for surfactin production (Cosby et al., 1998). The main obstacle to the commercialization of lipopeptides is also the setup of long-time production processes in bioreactors adapted to these highly foaming compounds (Coutte et al., 2017). The high foaming properties of these surface-active compounds cause a massive overflow of the culture broth out of the bioreactor, which often shortens the culture duration. Two main strategies have been investigated to face this issue: avoid foaming or favor foaming. Non-foaming culture processes were investigated with integrated bubbleless membrane bioreactors, rotating disks bioreactors, and biofilm bioreactors; see Coutte et al. (2017) for review. But for many reasons (production yield, process cost, and robustness), the upscale of these processes at a routine industrial level is not completed yet. It can be noticed that to avoid foaming, a classical manner is to add chemical antifoams in the culture broth, but this solution is not fully suitable here because antifoaming agents can have an inhibitory effect on the biomass physiology and interfere with oxygen transfer (Lee and Kim, 2004). Moreover, this strategy can complicate the separation and purification of the biosurfactant molecules, which interact with the antifoam agent. The addition of antifoam can also be detrimental for the analysis of the compounds of the culture medium using very sensitive analytical techniques such as NMR. Foaming culture processes coupled with foam separation were investigated for surfactant (Winterburn et al., 2011; Zhang et al., 2015) and lipopeptide production. The authors first optimized the foam overflowing rate by defining appropriate mixing and air supply conditions (Davis et al., 2001). But in this work, batch fermentation needed to be terminated in a short period of time, inferior to 36 h, because the fermentation broth was rapidly carried away by the overflowing foam. Similar processes were developed for the batch production of pseudofactins (Biniarz et al., 2020) or surfactins (Gong et al., 2009) with a fermenter coupled to foam collectors, but the duration of the culture could not exceed 40 and 32 h. An optimized design was proposed by adding a cell recycler and a surfactin precipitator which helped to keep the culture broth volume in the fermenter and allowed an effective batch fermentation time above 60 h (Yeh et al., 2006). In an effort to maintain the broth volume constant in the bioreactor to increase the duration and increase the overall productivity of the process, in particular by reducing the time for cleaning, sterilization, and handling of the vessels, the authors proposed the setup of fed-batch and continuous cultures. Exponentially fed-batch cultures were designed for mycosubtilin production (Guez et al., 2007; Chenikher et al., 2010), but serious difficulties in maintaining constant the volume of the broth upon 48 h were encountered (duration of the batch phase included). A more sustainable strategy was proposed with combining a continuous culture and foam fractionation for surfactin production (Chen et al., 2006). In these conditions, the production of surfactin was maintained over 100 h.

To overcome the limitation of the duration of the production process and also to facilitate the product recovery, a new process based on the well-described chemostat with feedback of biomass and dual effluent (Pirt and Kurowski, 1970) is proposed in the present work. It allows maintaining constant the reaction volume for sustainable pseudo-steady state culture conditions and permits the lipopeptide continuous removal through the collection of the overflowing foam. First, environmental conditions (medium composition and pH) were investigated in shake conical flasks to improve the production of mycosubtilin by the natural strain *Bacillus subtilis* American Type Culture Collection (ATCC) 6633 and limit the production of surfactin, another lipopeptide. Secondly, a bioprocess engineering approach was developed to set up the so-called overflowing continuous culture (O-CC) at the bioreactor scale. The specific geometric configuration of the agitation device of the bioreactor was fixed. The stirring was studied to ensure adapted foaming allowing a sustainable continuous culture process. Two mutants of *B. subtilis* ATCC 6633 were then cultivated in this O-CC bioprocess to evaluate the robustness of the process regarding different types of foam, which depends on the type of lipopeptide produced. The mycosubtilin specific productivity obtained during O-CC with a mycosubtilin constitutive overproducing strain BBG116, which also produces surfactin, was compared with the one obtained with a mycosubtilin constitutive overproducing strain BBG125, depleted for the surfactin synthesis.

## Materials and Methods

### Strains

*Bacillus subtilis* ATCC 6633 is a natural isolate producing two lipopeptides, i.e., mycosubtilin and surfactin. Its derivative BBG116 was obtained by replacing the native promoter P*_*myc*_* of the mycosubtilin synthetase operon with a constitutive promoter P*_*repU*_* originating from *Staphylococcus aureus* plasmid pUB110 (Béchet et al., 2013). *B. subtilis* BBG125 was obtained from BBG116 by interruption of the gene *srfAA*, the first gene of the *srfA* operon that codes for the surfactin synthesis. The strain description is presented in Table 1.

**TABLE 1:** Strains and plasmids.

Strain	Description	References
*B. subtilis* ATCC 6633	Produces mycosubtilin (Myc+), surfactin (Srf+), subtilin, subtilosin and rhizocticins	Duitman et al., 1999
*B. subtilis* BBG116	ATCC6633 derivative overproducing mycosubtilin; Myc + ⁣ + ⁣ + Srf+ Amy^–^ Spc^*R*^ Nm^*R*^; [P*repU-neo*::*myc*]	Béchet et al., 2013
*B. subtilis* BBG125	Mycosubtilin monoproducer BBG116 derivative; Myc++ Srf− Amy^–^ Spc^*R*^ Nm^*R*^ Cm^*R*^ Tc^*R*^; [P*repU-neo*::*myc*; *srfAA*::*cat-tet*]	Béchet et al., 2013

### Culture Media and Growth Conditions

#### In Shake Flasks

The preculture Clark medium (Clark, 1981) was modified by lowering the glucose concentration from 40 to 20 g⋅L^–1^ and inoculated with *B. subtilis* ATCC 6633 strain stored at −80°C in 40% (v/v) glycerol. The Clark preculture medium was composed of the following: glucose, 20 g⋅L^–1^; KH_2_PO_4_, 2,7 g⋅L^–1^; K_2_HPO_4_, 18,9 g⋅L^–1^; yeast extract, 0.5 g⋅L^–1^; EDTA, 0.05 g⋅L^–1^; MgSO_4_, 0.61 g⋅L^–1^; MnSO_4_, 0.056 g⋅L^–1^; NaCl, 0.1 g⋅L^–1^; CaCl_2_, 0.012 g⋅L^–1^; ZnSO_4_, 0.018 g⋅L^–1^; FeSO_4_, 0.018 g⋅L^–1^; CuSO_4_, 0.002 g⋅L^–1^; Na_2_MoO_4_, 0.001 g⋅L^–1^; H_3_BO_3_, 0.001 g⋅L^–1^; Na_2_SO_3_, 0.001 g⋅L^–1^; NiCl_2_, 0.0037 g⋅L^–1^; NH_4_NO_3_, 4 g⋅L^–1^; and MgSO_4_, 1 g⋅L^–1^. Cells were then transferred to 500-ml conical flasks with a filling volume of 100 ml of the culture medium. The initial biomass concentration for cultures was above 0.08 g(DW)⋅L^–1^, corresponding to an optical density (OD) at 600 nm of 0.25 (Kontron Uvicon 922). Cells were grown at 30°C with a shaking speed of 160 rpm and 50-mm shaking diameter. Different semi-synthetic culture media were tested: the Landy medium (Landy et al., 1948), the ammonium citrate sucrose (ACS) medium (Walton and Woodruff, 1949), and the Difco sporulation medium (DSM). The Landy medium contained the following: glucose, 20 g⋅L^–1^; glutamic acid, 5 g⋅L^–1^; yeast extract, 1 g⋅L^–1^; K_2_HPO_4_, 1 g⋅L^–1^; MgSO_4_, 0.5 g⋅L^–1^; KCl, 0.5 g⋅L^–1^; CuSO_4_, 1.6 mg⋅L^–1^; Fe_2_(SO_4_)_3_, 1.2 mg⋅L^–1^; and MnSO_4_, 0.4 mg⋅L^–1^. The ACS medium was composed of the following: sucrose, 100 g⋅L^–1^; citric acid, 11.7 g⋅L^–1^; yeast extract, 5 g⋅L^–1^; (NH_4_)_2_HPO_4_, 4.2 g⋅L^–1^_;_ Na_2_SO_4_, 4 g⋅L^–1^; KCl, 0.72 g⋅L^–1^; MgCl_2_, 6H_2_O, 0.42 g⋅L^–1^; ZnCl_2_, 10.4 mg⋅L^–1^; FeCl_3_, 6H_2_O, 24.5 mg⋅L^–1^; and MnCl_2_, 4H_2_O, 18.1 mg⋅L^–1^. The DSM medium contained bacto nutrient broth, 8 g⋅L^–1^; KCl, 1 g⋅L^–1^; MgSO_4_, 7H_2_O, 250 mg⋅L^–1^; Ca(NO_3_)_2_, 4H_2_O, 236 mg⋅L^–1^; MnCl_2_, 4H_2_O, 0.2 mg⋅L^–1^; and FeSO_4_, 7H_2_O, 0.03 mg⋅L^–1^. Cultures at different pH values were done in culture medium buffered with either 3-(*N*-morpholino)propanesulfonic acid (MOPS) or 2-(*N*-morpholino)ethanesulfonic acid (MES) at 100 mM. Experiments in conical flasks cultivations were carried out in triplicate; mean value and standard deviation were calculated.

#### In Stirred Tank Reactor

The medium chosen for the stirred tank reactor experiments was the Landy medium at a pH value of 6.5. A slight modification was done by reducing the concentration of glutamic acid from 5 to 2 g⋅L^–1^. A cheaper nitrogen source, ammonium sulfate, was added to balance correctly the medium without impact on mycosubtilin production. The Landy medium composition was modified as follows: glucose, 20 g⋅L^–1^; glutamic acid, 2 g⋅L^–1^, (NH_4_)_2_SO_4_, 2.3 g⋅L^–1^; yeast extract, 1 g⋅L^–1^; K_2_HPO_4_, 1 g⋅L^–1^; MgSO_4_ 0.5 g⋅L^–1^; KCl, 0.5 g⋅L^–1^; CuSO_4_, 1.6 mg⋅L^–1^; Fe_2_(SO_4_)_3_, 1.2 mg⋅L^–1^; and MnSO_4_, 0.4 mg⋅L^–1^. For each inoculum and culture in a bioreactor, *B. subtilis* BBG116 and BBG125 cells were taken from a −80°C frozen stock, and two precultures were done in a modified Landy medium. Stirred tank reactors used in this study were Bioflo 3000 (New Brunswick, NJ, United States) and EZ-Control (Applikon Biotechnology B.V., Netherlands) with both a nominal maximal working volume of 5 L. The bioreactor, containing 3 L of culture medium, was inoculated with early logarithmic grown cells and prewashed with one volume of sterile physiological water after a 5-min and 3,000-*g* centrifugation. The initial value of OD at 600 nm comprised between 0.1 and 0.2 u⋅OD. The temperature was 30°C, and the pH was controlled at the value of 6.5 by adding KOH (3 M) or H_2_SO_4_ (3 M) solutions. In order to have a precise regulation and avoid the degradation of the foam by titrants solutions, these additions were directly carried out on the surface of the culture medium thanks to a flush tube. The same technique was used for the feeding of the fresh substrate, thus limiting the impact of the foam on the dispersion of the substrate. The dissolved oxygen (DO) concentration was controlled above 15% of the saturation thanks to the adaptive stirrer speed. Aeration of the broth was ensured with insufflating 0.22-μm sterilized air at 0.25 VVM. The purity of the culture was tested on Mossel agar plates (Serva, Heidelberg, Germany). Culture medium, waste, and collected foam samples were taken under sterile working conditions.

### The Overflowing-Continuous Culture

#### Geometric Configuration of the Stirred Tank Reactor

Two Rushton impellers were fixed specifically on the rotating axis of the stirring device of the tank reactor (detailed Supplementary Figure 1 is given in the Supplementary Material section). The lower impeller was immersed in the culture broth as is usually done to ensure the mixing and oxygen transfer. The upper impeller was fixed just above the culture broth surface. The working volume of the bioreactor was fixed to 3 L. During the feeding phase of the process, the broth volume increased until the level of the upper impeller was reached, favoring the mixing at the gas–liquid interface. In these operating conditions, the impeller increased the foamability of the broth.

#### Setup of the Overflowing-Continuous Culture

##### Foamability Study

Foamability tests were done by adapting a previously described setup (Saint-Jalmes et al., 2005). The bubble column used by these authors was replaced by a stirred tank reactor. At the bottom of the tank, the air was blown at controlled rates through the sparger into the culture broth (constant bubble size). The air rate was fixed at 0.75 L⋅min^–1^. Considering the culture broth, the temperature was fixed at 30°C, the pH at 6.5, the concentration in biomass at 1.3 g(DW)⋅L^–1^. The mycosubtilin concentration was fixed at the maximal value that can be obtained during foam overflowing cultures, i.e., 2 mg⋅L^–1^. The foam volume *V*_*foam*_ was deduced from foam height measured as a function of time, with a time lapse of 5 min, and compared with the volume of gas injected *V*_*gas*_. The constant value of foamability *K* = *V*_*foam*_/*V*_*gas*_ was measured for different stirring speeds corresponding to different peripheral speeds of the Rushton turbine, calculated at the tip of the blade.

##### The Overflowing-Continuous Culture

**FIGURE 1:**
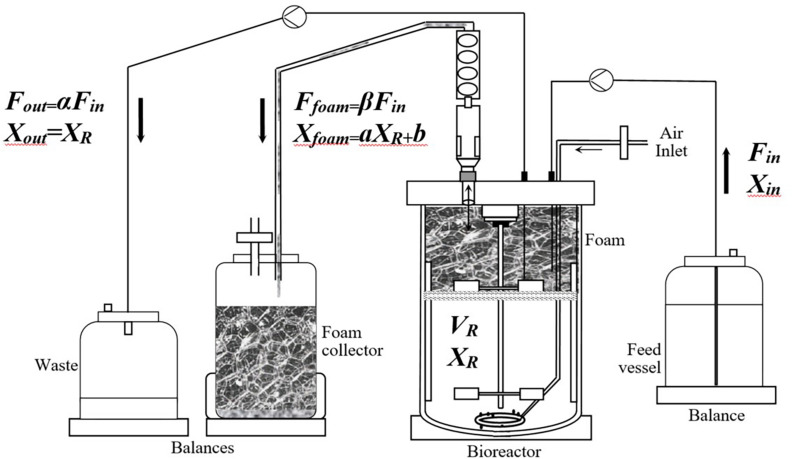
Flow sheet of the overflowing continuous culture (O-CC).

*Description of the Process*. The term “overflowing continuous culture” as used in this paper describes an original continuous culture process whereby the outgoing outflow rate is divided into two separate flow rates, the outgoing flow rate in the waste vessel (*F*_*out*_) and the foam overflowing flow rate (*F*_*foam*_), as shown in Figure 1.

**FIGURE 2:**
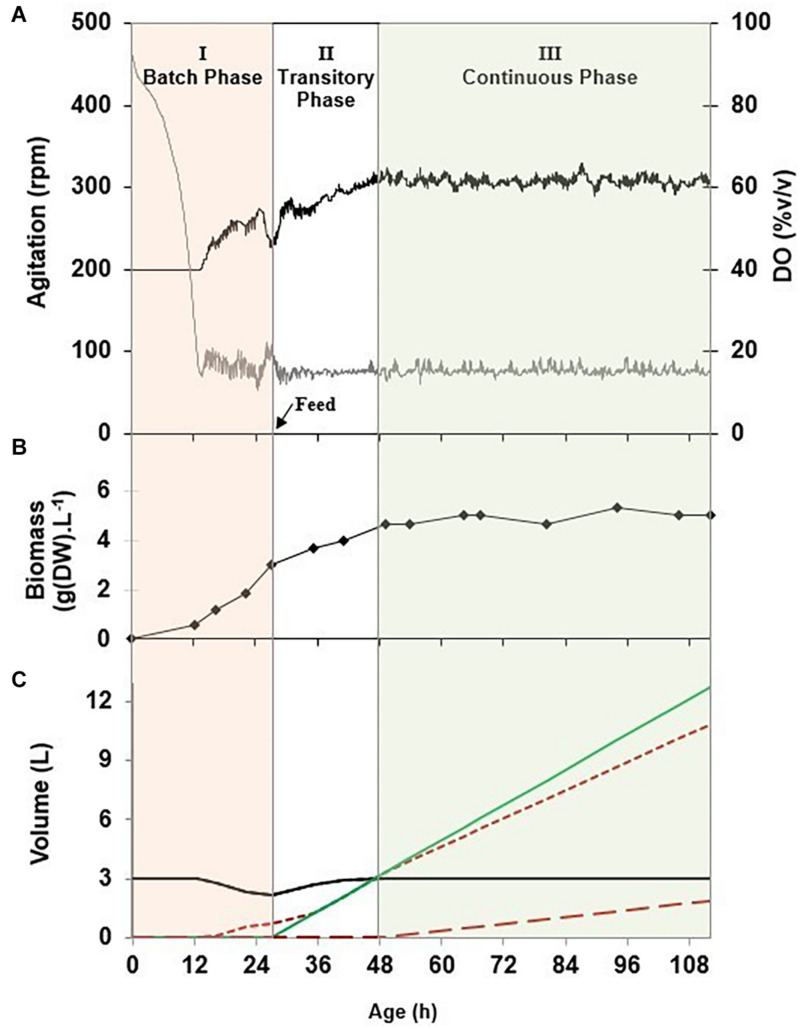
**(A)** Evolution upon time of the stirrer speed (^___^) and the dissolved oxygen (^___^) during an overflowing continuous culture (O-CC) of *Bacillus subtilis* BBG116 in the modified Landy culture medium at a temperature of 30°C, a pH of 6.5 u⋅pH, a dilution rate of 0.05 h^–1^, and a dissolved oxygen value regulated above 15% v/v with an agitation cascade mode of between 200 and 400 rpm. **(B)** Evolution of the biomass dry weight (◆) in the stirred tank reactor. **(C)** Evolution of the volumes of the stirred tank reactor *V*_*R*_ (^___^), the feed (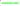), the collected foam (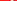), and the waste (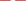).

The process was separated into three successive phases exemplified in Figure 2. In phase I, cells were grown in a batch culture at the maximal specific growth rate, i.e., 0.33 h^–1^. DO was controlled above 15% v/v with an adaptive stirring strategy. The phase I duration comprised between 24 and 28 h, until the observation of a decrease of the stirrer speed and a rise of the DO value, expressing a limitation in a substrate. In this phase, the volume of liquid remaining in the tank reactor decreased and the volume of the foam collected increased. This phenomenon was observed because of the foam overflowing flow rate occurring during the continuous production of mycosubtilin due to the constitutive P*_*repU*_* promoter inserted in *B. subtilis* ATCC 6633 derivatives, BBG116 and BBG125. It has to be noticed that in the case of BBG116, which has a (Myc+++ Srf+) phenotype, the foam overflowing flow rate was also linked to the residual production of surfactin.

Phase II could be described as a transitory feed phase that aimed at returning to the initial broth volume in the tank reactor, which allowed the management of the foaming capacity of the broth as described at section “Geometric Configuration of the Stirred Tank Reactor”. For experiments performed in a 3-L bioreactor at a dilution rate comprised between 0.05 and 0.1 h^–1^, the feed flow rate of phase II comprised between 150 and 300 ml⋅h^–1^. As the overflowing foam of phase I corresponded to a volume loss comprised between 300 and 900 ml, the return to the initial broth volume took at least between 1 and 4 h. This duration was increased because the foam overflowing flow rate occurring during phase I also occurred during phase II. To gain time and return faster to the initial broth volume, the feed flow rate of phase II could be increased as long as it corresponded to a value of the dilution rate below the value of the washout rate of the microorganism, i.e., 0.33 h^–1^.

Phase III corresponded specifically to the so-called O-CC phase. The feeding strategy was applied in reference to a continuous culture protocol with a given dilution rate as a reference parameter. The originality of the present process came from the outgoing flow rate that was divided into two separate flows: the outgoing flow rate in the waste vessel (*F*_*out*_) and the foam overflowing flow rate (*F*_*foam*_). For the setup of a long duration and sustainable foam overflowing process, it was indeed necessary to allow the pumping out of the tank reactor of the broth because it was observed that the volume of the broth in the reactor could increase punctually beyond the broth volume operating point of 3 L. This phenomenon was linked to *F*_*foam*_ that encountered slight variations over time. Pumping out the broth when its volume increased beyond the operating point was found to be an efficient strategy to keep constant the broth volume in the tank. In these conditions, the sum of the overflowing foam and the outgoing flow rates was equal to the feed flow rate.

*Modeling of the Biomass During Overflowing-Continuous Culture and Theoretical Aspects*. In phase III of the O-CC, the biomass change in the stirred tank reactor could be expressed as follows:

(1) $$V_{R}\cdot d\,X_{\text{R}}/d\,t=X_{i\,n}\cdot F_{i\,n}+V_{R}\cdot \mu \cdot X_{R}-X_{R}\cdot F_{o\,u\,t}-X_{f\,o\,a\,m}\cdot F_{f\,o\,a\,m}$$

The process conditions of phase III were as follows:

(2) $$F_{o\,u\,t}=\alpha \,F_{i\,n}$$

(3) $$F_{f\,o\,a\,m}=\beta \,F_{i\,n}$$

with *F*_*in*_ = *F*_*out*_ + *F*_*foam*_ (4), expressing the constant volume operation. In these conditions, α + β = 1.

The modeling of the biomass extraction by the foam led to the following:

(4) $$X_{f\,o\,a\,m}=X_{R}+b$$

The biomass extraction model in the foam overflow was written with three main assumptions:

(a) *X*_*foam*_ proportional to *X*_*R*_ (*a* ≤ 1 and *b* = 0), corresponding to a partial recycling process.
(b) *X*_*foam*_ equal to *X*_*R*_ (*a* = 1 and *b* = 0), corresponding to a continuous one, without recycling.
(c) *a* < 1 and *b*≠0, corresponding to the foaming process (Guez et al., 2007).

Taking into account equations (2–4), equation (1) could be rewritten as follows:

(5) $$V_{R}\cdot d\,X_{\text{R}}/d\,t=X_{i\,n}\cdot F_{i\,n}+V_{R}\cdot \mu \cdot X_{R}-X_{R}\cdot \alpha \cdot F_{i\,n}-\left(\alpha \,X_{R}+b\right)\cdot \beta \cdot F_{i\,n}$$

During phase III, the biomass concentration in the tank was shown to be pseudo constant, so *dX*_*R*_/*dt* = 0; and the concentration of biomass in the feed vessel was X=i⁢n0 g⋅L^–1^. In steady state, $D=\frac{F_{i\,n}}{V_{R}}$, so that equation (1) could be rewritten and the microorganism growth rate could finally be expressed as follows:

(6) $$\mu =D\,\left(\alpha +a\,\beta +\frac{b\,\beta }{X_{R}}\right)$$

For a mean value of the concentration of biomass in the tank, *X_*R*__*mean*_*, a mean value of the specific growth rate, μ*_*mean*_*, could be computed.

### Analysis

#### Lipopeptide High-Performance Liquid Chromatography Analysis

The lipopeptide analysis protocol was derived from previous work (Guez et al., 2007; Béchet et al., 2013). Culture and overflowed foam samples were centrifuged at 10,000 *g* for 10 min. The sample was first diluted in 50% EtOH, and 20 μl was then injected and analyzed by high-performance liquid chromatography using a C18 column (5 μm, 250 × 3.0 mm, 218 TP, Vydac, Grace) on Acquity UPLC system (Waters, Milford, MA, United States). Mycosubtilins were separated with an acetonitrile/water/trifluoroacetic acid (TFA) solvent, 40:60:0.1 v/v/v during 20 min, and surfactins with an acetonitrile/water/TFA 80:20:0.1 v/v/v during 20 min. The flow rate was 0.6 ml⋅min^–1^, and detection wavelength was 214 nm. Quantification was performed by adding the areas of the peaks identified as lipopeptides and then transferring this total area to a calibration curve obtained with standard molecules between 10 and 1,000 mg⋅L^−1^. Purified iturin A and surfactins used as standards were from Sigma Aldrich (Saint Louis, United States). The retention time and second derivative of the absorption spectrum between 200 and 400 nm were used to identify the eluted molecules (Empower Software, Waters). For the analysis of flask experiments, samples were first concentrated using the following method. A volume of 1 ml of the supernatants was purified through C18 Maxi-Clean cartridges (Alltech, Deerfield, United States). The charged column was washed with 8 ml of water. The lipopeptides were then eluted with 6 ml of 100% methanol [high-performance liquid chromatography (HPLC) grade, Acros Organics, Geel, Belgium]. The extract was brought to dryness before dissolution in 200 μl of methanol. Examples of iturin A standard as well as mycosubtilin and surfactin chromatograms of samples taken from shake flasks experiments were annotated and presented in Supplementary Figure 2.

#### Lipopeptide Tandem Mass Spectrometry Analysis

The determination of the different homologs of mycosubtilin produced by the natural strain ATCC 6633 and its derivative BBG116 and BBG125 was achieved by analyzing purified samples by tandem mass spectrometry (MS–MS) with electrospray and ion trap. Measurements were made by direct infusion of the open peptide after treatment with *N*-bromosuccinimide (Peypoux et al., 1981, 1986). The dry peptide was treated with 20 μl of a solution containing an equal amount of *N*-bromosuccinimide in 70% acetic acid for 3 h at room temperature. The excess of reagent was destroyed by 5 μl of formic acid. The hydrolysate was evaporated to dryness. Time-dependent MS–MS was made with a Bruker Esquire HCT electrospray ionization (ESI)–mass spectrometer. The conditions of the electrospray were as follows: 180 μl⋅h^–1^ flow rate; ultrascan mass range mode; positive ion polarity; ESI source; 300°C dry temperature; 15 psi of N_2_ at nebulizer; 4 L⋅min^–1^ of dry gas; and 4,000 V for high-voltage (HV) capillary.

#### Bacterial Dry Weight Analysis

The bacterial dry weight was determined after drying 48 h at 110°C a washed pellet of a 10-ml sample. The OD at 600 nm was read with a spectrophotometer (UV Mini 1 240, Shimadzu, Japan). An OD of 1.0 corresponded to a biomass concentration of 0.33 g(DW)⋅L^–1^ in the Landy medium.

## Results and Discussion

### Environmental Study for Increasing the Productivity and the Selectivity of Mycosubtilin Biosynthesis

#### Effect of the Culture Medium

Lipopeptide production is largely dependent on the composition of the culture medium. The authors have shown this dependency for iturins. Glucose, fructose, and mannitol were identified as the best carbon sources (Besson and Michel, 1987). Amino acids such as L-glutamic acid, L-proline, L-valine, and L-glutamine were shown to be suitable for the production of iturin A by *Bacillus subtilis* (Sandrin et al., 1990). In contrast, L-isoleucine, L-tryptophan, and L-lysine did not promote the biosynthesis of iturin A. In this study, three different media were investigated for the synthesis of mycosubtilin by *B. subtilis* ATCC 6633. These media are known to be suitable for lipopeptide production: the Landy medium (Landy et al., 1948), the ACS medium (Walton and Woodruff, 1949), and the DSM (Nakano et al., 1988).

In the Landy medium, the lipopeptide production by *B. subtilis* ATCC 6633 was equal to 49.0 ± 9.7 mg⋅L^–1^ (see Table 2). The ACS medium allowed a sensibly larger amount of lipopeptide production. The DSM medium did not allow the production of mycosubtilin. The selectivity of the lipopeptide production was illustrated by the mycosubtilin/surfactin ratio. As presented in Supplementary Figure 2, the lipopeptide analysis showed four major isoforms of mycosubtilin, from C16 to C17, and six major isoforms of surfactin from, C13 and/or C14 to C15 and/or C16 (the random presence of Leu, Val, or Ile in position 7 associated with different lengths of the fatty acid moiety makes mass spectrometry analysis for surfactin). Results showed that the Landy medium helped to orientate the lipopeptide synthesis rather toward mycosubtilin than surfactin. The mycosubtilin specific productivity of 0.228 mg⋅g(DW)^–1^⋅h^–1^ obtained with the Landy medium was higher than the one obtained with the ACS medium. The maximal biomass yield on glucose was equal to 0.23 g(DW)⋅g^–1^ for experiments done in the Landy medium, close to the value reported previously for similar culture conditions (Guez et al., 2007) but still lower than values generally reported for cultures of *Bacillus* in the presence of glucose as carbon source. In the Landy medium, a probable energetic loss occurs due to the production of acetate (up to 0.5 g⋅L^–1^) and the consumption of glutamic acid, which leads to the non-oxidative conversion of pyruvic acid to acetoin (Keynan et al., 1954). The maximal biomass yield on glucose was not computable for DSM medium (absence of glucose) and ACS medium (glucose not depleted after 48 h of culture due to the high sucrose initial concentration of 100 g⋅L^–1^). Taken together, these results showed that the Landy medium was more suitable than the other media to achieve a productive and selective synthesis of mycosubtilin. The different effects of the medium on the production of mycosubtilin and surfactin indicate different regulations of their synthesis pathways. The authors had previously shown that the lipopeptides synthesized by ATCC 6633 were not regulated by the same mechanisms (Duitman et al., 2007). It is indeed known that ComA modulates *srfA* operon expression, coding the surfactin synthetase, but not the *myc* operon expression, coding the mycosubtilin synthetase. The maximal pH variation Δmax of 2.8 pH units obtained with the Landy medium was the result of strong acidification of the culture broth at the beginning of the exponential growth phase followed by an alkalinization step. However, the high amplitude of the pH fluctuations should be limited.

**TABLE 2:** Effect of the Landy, ACS, and DSM media on the biomass dry weight concentration, on the pH (culture start, culture end, and maximal pH variation), on mycosubtilin and surfactin concentrations, on mycosubtilin specific productivity, and on the mycosubtilin/surfactin ratio.

Medium	Biomass	pH			Mycosubtilin	Surfactin	*q*_P_*	Ratio
	g(DW)⋅L^–1^	Start	End	Δmax	mg⋅L^–1^	mg⋅L^–1^	mg⋅g^–1^⋅h^–1^	
Landy	4.78 ± 0.07	7.0	7.8	2.8	49.0 ± 9.7	1.0 ± 0.5	0.228	49
ACS	8.70 ± 0.06	7.0	7.2	1.2	66.1 ± 9.1	15.2 ± 5.2	0.165	4
DSM	1.36 ± 0.01	6.9	9.2	2.3	1.0 ± 0.5	1.0 ± 0.5	0.022	1

Cultures of the natural strain Bacillus subtilis ATCC 6633 were done for 48 h. Measurements are given as means from triplicates of independent cultivations with standard deviation. ACS, ammonium citrate sucrose; DSM, Difco sporulation medium; ATCC, American Type Culture Collection. *Calculated for mycosubtilin.

#### Effect of the pH

Growth of *B. subtilis* strains in the Landy medium was firstly characterized by strong acidification of the culture medium due to organic acid synthesis, principally acetate and lactate as previously demonstrated (Coutte et al., 2013), followed by an alkalinization phase correlated to the consumption of these organic acids and of the glutamic acid initially present in the medium (data not shown). In a flask, such pH variation can be avoided or limited by using zwitterionic buffers like MES or MOPS, which are not consumed by the strain.

**FIGURE 3:**
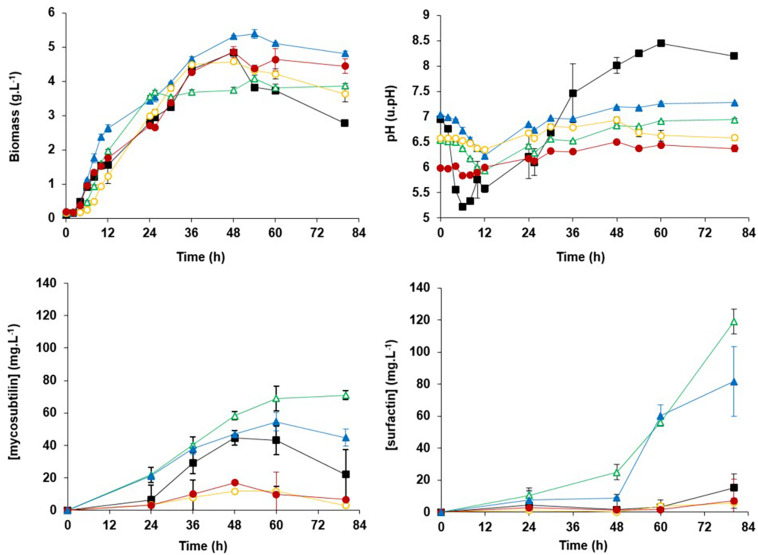
Biomass, pH, production of mycosubtilin, and surfactin kinetics for *Bacillus subtilis* American Type Culture Collection (ATCC) 6633 flasks cultures at 30°C in the Landy medium (■) or in the Landy medium buffered with 100 mM of 3-(*N*-morpholino)propanesulfonic acid (MOPS) at pH 7.0 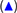 or at pH 6.5 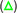 or with 100 mM of 2-(*N*-morpholino)ethanesulfonic acid (MES) at pH 6.5 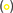 or at pH 6.0 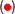. Measurements are given as means from triplicates of independent cultivations with standard deviation.

An experimental study was performed through the cultivation of *B. subtilis* ATCC 6633 in Landy medium set at different pH values with these zwitterionic buffers. Results showed that the variation of pH was sufficiently reduced from 2.5/3.0 units without a buffer to 0.8/0.9 and 0.6/0.7 in the presence of MOPS and MES, respectively (Figure 3). In these buffer conditions, the kinetic of the microorganism growth and the production of mycosubtilin and surfactin was studied.

Mycosubtilin production reached 69 mg⋅L^–1^ after 60 h of culture in the Landy medium buffered with MOPS 100 mM at a pH initial value of 6.5, better than the production reached with MOPS 100 mM at a pH initial value of 7.0. This production was also sixfold higher than the production observed when using the MES buffer at the same initial pH value of 6.5. The difference in mycosubtilin production obtained with two different buffers set at the same initial pH value can have two origins. First, it can be explained by the variation of the pH during the culture that is different between the two experiments because of the different pK_*a*_ values of MOPS and MES (7.2 and 6.1, respectively). Second, it was reported that MES buffer or its oligo(vinyl succinic acid) contaminants could interfere with cellular metabolisms (Vilarino et al., 1997). To determine if the strong negative effect of MES was linked to the buffer effect or a potential inhibition of the lipopeptide synthesis, an experiment was carried out in pH-controlled stirred tank reactor in the presence or not of MES. The results (data not shown) confirmed the inhibitory effect of MES on lipopeptide biosynthesis.

In the Landy medium buffered with MOPS 100 mM at pH 6.5, no decrease of biomass and mycosubtilin productions was observed. In all other culture conditions, a drop of the biomass and the mycosubtilin production was observed at the beginning/middle of the stationary phase, after the depletion of carbon substrate, more likely linked to cellular lysis and mycosubtilin hydrolysis. A decrease in the concentration in lipopeptide was previously reported during surfactin production processes (Guez et al., 2008). It could be explained with the highlighting of surfactin-specific hydrolase activities (Grangemard et al., 1999; Hoefler et al., 2012), but no evidence has been given by now for the hydrolysis of mycosubtilin.

The surfactin production during cultures remained below 10 mg⋅L^–1^ for all conditions tested in the first 24 h. It remained below 15 mg⋅L^–1^ until the end of the culture for the experiments without buffer and with MES buffer. In contrast, the surfactin production remained below 25 mg⋅L^–1^ until 48 h of culture for experimental conditions with MOPS buffer. After 48 h of culture, the production increased continuously and reached 80 and 120 mg⋅L^–1^ with MOPS at pH 7.0 and MOPS at pH 6.5, respectively. Moreover, the best productivity and selectivity (mycosubtilin/surfactin ratio) are obtained with MOPS 100 mM at pH 6.5. Taken together, these results led us to choose the Landy medium at pH 6.5. buffered with MOPS 100 mM.

### Overflowing-Continuous Culture Setup

#### Foamability Optimization

Foamability was investigated to define an appropriate stirring range allowing a sufficient foam overflowing flow rate during O-CC processes. Experiments performed at peripheral speeds of the tip of the Rushton turbine of 0.40, 0.80, 1.00, and 1.20 m⋅s^–1^ have achieved a constant value of the foam height corresponding, respectively, to a foamability K of 0.15, 0.30, 0.48, and 0.60. According to the authors (Saint-Jalmes et al., 2005), K values lower than 0.1 represent a low foamability. In our application, the goal is to promote foaming. Thus, it was decided to fix the minimum peripheral speed to 0.80 m⋅s^–1^, providing a minimal K value of 0.30. An additional experiment conducted at 1.60 m⋅s^–1^ (data not shown) did not allow the stabilization of the foam height in the experimental time. The foamability value could not be calculated for this condition, but the volume of foam/volume of air injected tends to a K value of 0.97. Since the stabilization of the foam height in the tank at a peripheral speed of 1.60 m⋅s^–1^ could not be observed, we preferred to choose a maximum peripheral speed of 1.20 m⋅s^–1^, providing a K value of 0.60. The range of agitation fixed for the O-CC process was thus fixed between 0.80 and 1.20 m⋅s^–1^, ensuring K values ranging from 0.30 to 0.60.

#### Overflowing-Continuous Culture

The mycosubtilin constitutive overproducer mutant BBG100 (Myc+++ Srf+) (Leclere et al., 2005), which was utilized previously for the setup of exponentially fed-batch cultures (Guez et al., 2007), was shown to be slightly unstable. The construction of a novel mutant was therefore undertaken (Béchet et al., 2013), leading to the isolation of a stable mycosubtilin overproducing strain named BBG116 (Myc+++ Srf+). Transformation of BBG116 with knocking-out *srfAA* surfactin synthetase gene led to the isolation of a mycosubtilin monoproducer BBG125 (Myc++ Srf−). BBG125 was shown to synthesize slightly less mycosubtilin than its parent BBG116. These strains were tested within the setup of long duration and sustainable O-CC for two main reasons. First, these strains were more stable over time in comparison with the previously tested BBG100 strain. Second, the production of lipopeptide was different, as BBG125 did not produce any surfactin. As the foaming properties of the broth depend on the amphiphilic structure and molecular size of the produced surface-active agents (Razafindralambo et al., 1998), it appeared relevant to test both strains within the O-CC process. It allowed testing its effect on the biomass extraction by foam. Five O-CC experiments using BBG116 and two more experiments using BBG125 were performed at different dilution rates comprised between 0.5 and 0.1 h^–1^.

##### Lipopeptide Recovery

For each experiment, the concentration of lipopeptides was measured in the stirred tank reactor, in the foam collector, and in the waste vessel, as described in Figure 1. For the BBG116 mutant (Myc+++ Srf+), both mycosubtilin and surfactin were found exclusively in the foam collected outside the bioreactor. For the BBG125 mutant (Myc++ Srf−), no surfactin was produced as expected, and mycosubtilin was also only found in the foam collector. The results of specific productivity in mycosubtilin are given in the sequel of this work. For both strains, the product recovery yield was superior to 99% similar to that obtained previously for mycosubtilin (Guez et al., 2007). Slightly lower results with comparable extraction techniques were previously described for surfactin (Davis et al., 2001; Willenbacher et al., 2014). Compared with routine downstream processing strategies of lipopeptides (Inès and Dhouha, 2015; Coutte et al., 2017), which can rapidly become time-consuming and can mobilize specific techniques (mainly acid or ammonium sulfate precipitation, solvent extraction, membrane ultrafiltration, and foam fractionation), the O-CC foaming process presented real advantage for *in-situ* product removal, facilitating eventual subsequent purification steps by reducing the volume of liquid to be purified (in our process conditions, this volume reducing factor was calculated at 1.82 ± 0.15).

##### Biomass Concentration

In the stirred tank reactor, the biomass concentration value was measured for samples taken during phase III of each O-CCs. Mean biomass concentration values of 4.83 ± 0.65, 7.29 ± 0.77, and 6.20 g⋅L^–1^ were calculated for experiments performed at dilution rates of 0.05, [0.071; 0.075] and 0.1 h^–1^. According to partial recycling fermentor theory (Pirt and Kurowski, 1970), these constant values of the biomass concentration measured for given dilution rates during phase III of the O-CCs showed that a pseudo-steady state could be reached in phase III. The mean biomass concentration value of 6.20 g⋅L^–1^ obtained at a dilution rate of 0.1 h^–1^ was lower than the one obtained at 0.075 h^–1^. This result could be explained by the assumption of a slightly lower biomass yield on glucose at a dilution rate of 0.1 h^–1^ compared with 0.075 h^–1^. This phenomenon linked to the physiology of *Bacillus*, reported previously by the authors (Rodríguez-Monroy, 1996).

**FIGURE 4:**
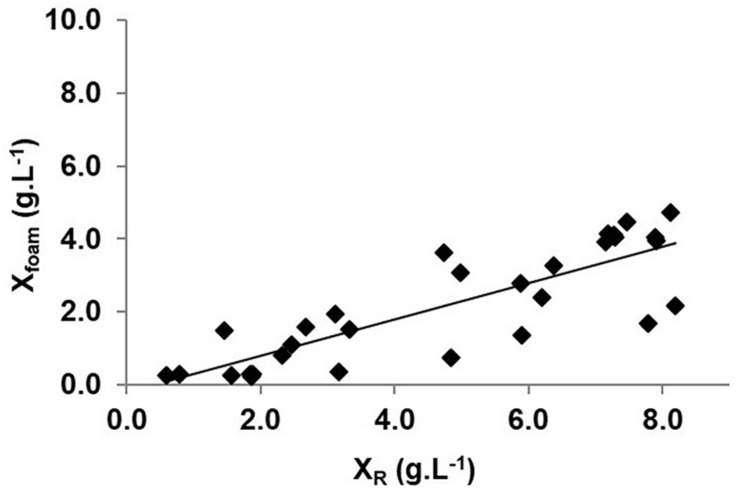
Evolution of biomass concentration in the foam (*X*_*foam*_) as a function of biomass concentration in the stirred tank reactor (*X*_*R*_) expressed in g(DW)⋅L^–1^.

In order to establish the relationship between the biomass concentration in the bioreactor and in the foam, measurements of biomass were done during the three phases of the process. Figure 4 exhibits a pseudo-linear relationship between these two parameters for values comprised between 0.5 and 8.0 g(DW)⋅L^–1^. Linear regression gives *a* = 0.50 and *b* = −0.21 g⋅L^–1^, which are two important model parameters defined in equation (6). It validates assumption (c) of the biomass extraction model given at “Modeling of the Biomass During Overflowing-Continuous Culture and Theoretical Aspects” section and indicates that the biomass concentration in the foam *X*_*foam*_ is dependent on the biomass concentration in the bioreactor *X*_*R*_. As *b* was close to 0 and a was inferior to 1, the O-CC process could be related to a continuous one with biomass feedback (Pirt and Kurowski, 1970). These results show a certain dispersion (R^2^ = 0.70) due to discrepancies in the determination of *X*_*foam*_, mainly explained by the sampling procedure, where foam is sampled in a short period disconnecting the bioreactor off-gas tube from the foam collector. The difficulty to measure *X*_*foam*_ accurately was already reported by the authors (Guez et al., 2007; Chenikher et al., 2010). It could also be explained by variations of the foam properties due to the presence of lipopeptides belonging to different families. In the case of O-CC processes operated with BBG116 strain, a remaining production of surfactin was indeed observed. Variations in the length of the alkyl chain of lipopeptides could also modify drastically the density and the stability of the foam as it was previously demonstrated in the case of iturin A, structurally close to mycosubtilin (Razafindralambo et al., 1998). In stirred tank reactor experiments, the majority of lipopeptides were mycosubtilins, which were shown to be present mainly as long-chain alkyl isoforms, composed of 19 ± 1% of mycosubtilin *iso*C16, 23 ± 2% of mycosubtilin *iso*C17, and 53 ± 2% of mycosubtilin *anteiso*C17 and minor isoforms. As this composition did not vary significantly over time, it could only partially be at the origin of changes in the foam properties. However, half of the biomass in the stirred tank reactor was entrained by foam during the process.

##### Correlation Between Mean Specific Growth Rate and Mycosubtilin Specific Productivity

**FIGURE 5:**
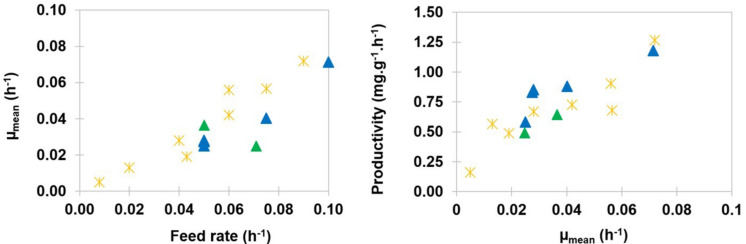
Mean growth rate *μ_*mean*_* as a function of the dilution rate expressed in h^–1^ (left). Specific productivity *q*_*P*_ expressed in mg of mycosubtilin⋅g(DW)^–1^⋅h^–1^, as a function of mean growth rate *μ_*mean*_* expressed in h^–1^ (right). Results are presented for strains BBG116 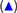 and BBG125 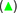. Results are presented with those obtained with BBG100 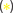 during previous exponentially fed-batch culture experiments (Guez et al., 2007).

The mean values of specific growth rates μm*_*ean*_* were calculated according to equation (6). They were found to be lower than the values of the corresponding dilution rates, which is typical from processes with dual effluent systems and feedback of biomass (Pirt and Kurowski, 1970). Figure 5 (left side) shows that the mean value of the specific growth rate could be approximated with a linear function of the dilution rate. This result was consistent with data reported with the strain BBG100 (Guez et al., 2007).

Specific productivity *q*_*P*_ of mycosubtilin could be presented as a function of the mean specific growth rate *μ_*mean*_* (Figure 5, right side). Within the range of feed rates that were tested, results showed that the productivity could also be approximated by a linear relation with the mean growth rate. Therefore, the maximum productivity value measured for the O-CC experiments, i.e., 1.18 mg of mycosubtilin g(DW)^–1^ of biomass h^–1^, corresponded to the maximum dilution rate tested in this work, i.e., 0.1 h^–1^. The values obtained for BBG116 were close to those obtained with BBG100 during previous exponentially fed-batch cultures. As expected (Béchet et al., 2013), the values obtained for BBG125 appeared to be slightly inferior. The O-CC process was maintained in these laboratory conditions until 148 h (batch time included). Compared with exponentially fed-batch cultures maintained for a maximal duration of 48 h (batch time included) (Guez et al., 2007), and considering that the cleaning and sterilization procedure takes 24 h, the mycosubtilin production rate was increased by a 2.06 factor with this new O-CC process. It could also be mentioned that the upscale of this process was performed successfully in 300 L at a semi-industrial scale.

## Conclusion and Prospects

Compared with other similar foaming processes, O-CC appeared to be a powerful tool for producing a surfactant compound in a stirred and aerated tank reactor; its major advantage lies in the possibility of making the process last over time, to increase the overall production rate. A new continuous process was thus modeled and set up, allowing the sustainable production and the total continuous removal of biosurfactants in bioreactor while recycling partially biomass and reducing the processing volumes handled for further downstream processes. An interesting prospect that would help to simplify the O-CC setup for industrial operators could consist in limiting the outgoing outflows to a single overflowing foam rate (*F*_*foam*_), meaning that the pumped withdrawn flow (*F*_*out*_) would not be necessary anymore. Fulfilling this purpose would require an increase of the foaming of the broth that could be obtained with optimized equipment (specific foaming impeller, dedicated stirred tank reactor, etc.) or a higher mycosubtilin production rate or by specifically studying the foam physico-chemical properties to better control its foamability. To intensify the O-CC process, this optimization work should also lead in parallel to the definition of the foam parameters (density and stability) aiming at lowering biomass entrainment in the overflowing foam. Finally, this process should also contribute to a facilitated coupling of foam fractionation tools, already developed for other lipopeptide biosurfactant production (Willenbacher et al., 2014; Alonso and Martin, 2016).

## Data Availability Statement

The raw data supporting the conclusions of this article will be made available by the authors, without undue reservation.

## Author Contributions

J-SG and FC conceptualized the experiments. J-SG, AV, and FC took part in the investigation, methodology, and analysis. J-SG wrote the manuscript and supervised the O-CC project. AV, CL, PJ, and FC helped in the critical review and the editing of the manuscript. PJ and FC participated in the funding acquisition. All authors contributed to the article and approved the submitted version.

## Conflict of Interest

FC and PJ from the University of Lille and University of Liège are also the two co-funders of Lipofabrik company which markets mycosubtilin and other lipopeptides from *B. subtilis*. J-SG from University Clermont Auvergne is an associate of Lipofabrik company. The remaining authors declare that the research was conducted in the absence of any commercial or financial relationships that could be construed as a potential conflict of interest.

## Supplementary Material

Supplementary Figure 1 Geometrical factors expressed in cm of the stirred tank reactor. D_*i*_ = 7,65; H_*L*_/D_*i*_ = 1,83; D_*t*_/D_*i*_ = 2,22; H_0_/D_*i*_ = 1,00; W_*i*_/D_*i*_ = 0,20; L_*i*_/D_*i*_ = 0,25.

Supplementary Figure 2 Annotated chromatograms of **(A)** iturin A standard, **(B)** mycosubtilin and **(C)** surfactin from shake flasks experiments. A volume of 20 μL was injected and analyzed using a C18 column (5μm, 250 × 3.0 mm, 218 TP, VYDAC, Grace) on Acquity UPLC system (Waters, Milford, MA, United States). Mycosubtilins (and iturin A standard) were eluted with a ACN/H_2_O/TFA solvent, 40:60:0.1, V/V/V, and surfactins with a ACN/H_2_O/TFA 80:20:0.1 solvent, V/V/V. The flow rate was 0.6 mL⋅min^–1^, detection wavelength was 214 nm. The retention time and second derivative of the absorption spectrum between 200 and 400 nm were presented above the chromatograms.

## Abbreviations

*F*_*in*_: feeding flow rate (L ⋅ h^–1^)
*F*_*foam*_: foam overflowing flow rate (L ⋅ h^–1^)
*F*_*out*_: outgoing flow rate in the waste vessel (L ⋅ h^–1^)
*V*_*R*_: volume of culture medium in the stirred tank reactor (L)
*V*_*foam*_: liquid volume in the foam collector (L)
*V*_*out*_: liquid volume in the waste vessel (L)
*X*_*R*_: biomass concentration in the stirred tank reactor (g ⋅ L^–1^)
*X*_*foam*_: biomass concentration in the foam (g ⋅ L^–1^)
*q*_*P*_: mycosubtilin specific productivity [mg ⋅ g^–1^(biomass DW) ⋅ h^–1^].
