# Supplementary figures and images for: New Continuous Process for the Production of Lipopeptide Biosurfactants in Foam Overflowing Bioreactor
Source: Front Bioeng Biotechnol. 2021 May 28;9:678469. doi: 10.3389/fbioe.2021.678469 (PMC8194703; doi:10.3389/fbioe.2021.678469)

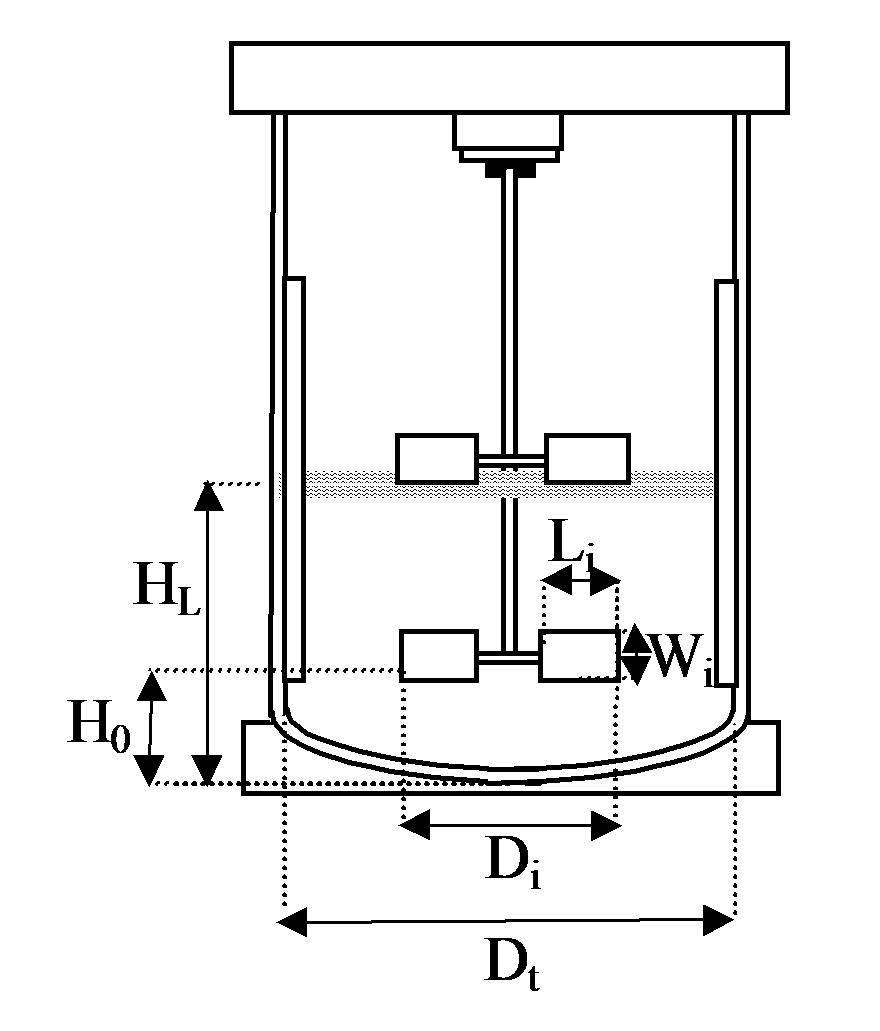

Supplement: Supplementary Figure 1 — Geometrical factors expressed in cm of the stirred tank reactor. Di = 7,65; HL/Di = 1,83; Dt/Di = 2,22; H0/Di = 1,00; Wi/Di = 0,20; Li/Di = 0,25. [file Image_1.JPEG]

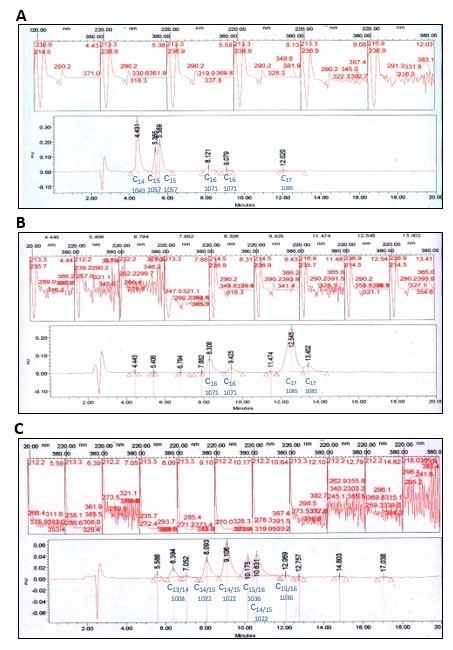

Supplement: Supplementary Figure 2 — Annotated chromatograms of (A) iturin A standard, (B) mycosubtilin and (C) surfactin from shake flasks experiments. A volume of 20 μL was injected and analyzed using a C18 column (5μm, 250 × 3.0 mm, 218 TP, VYDAC, Grace) on Acquity UPLC system (Waters, Milford, MA, United States). Mycosubtilins (and iturin A standard) were eluted with a ACN/H2O/TFA solvent, 40:60:0.1, V/V/V, and surfactins with a ACN/H2O/TFA 80:20:0.1 solvent, V/V/V. The flow rate was 0.6 mL⋅min–1, detection wavelength was 214 nm. The retention time and second derivative of the absorption spectrum between 200 and 400 nm were presented above the chromatograms. [file Image_2.JPEG]
